# Maximizing the Information Content of Experiments in Systems Biology

**DOI:** 10.1371/journal.pcbi.1002888

**Published:** 2013-01-31

**Authors:** Juliane Liepe, Sarah Filippi, Michał Komorowski, Michael P. H. Stumpf

**Affiliations:** 1 Centre for Integrative Systems Biology and Bioinformatics, Imperial College London, London, United Kingdom; 2 Institute of Fundamental Technological Research, Polish Academy of Sciences, Warsaw, Poland; 3 Institute of Chemical Biology, Imperial College London, London, United Kingdom; University of Chicago, United States of America

## Abstract

Our understanding of most biological systems is in its infancy. Learning their structure and intricacies is fraught with challenges, and often side-stepped in favour of studying the function of different gene products in isolation from their physiological context. Constructing and inferring global mathematical models from experimental data is, however, central to systems biology. Different experimental setups provide different insights into such systems. Here we show how we can combine concepts from Bayesian inference and information theory in order to identify experiments that maximize the information content of the resulting data. This approach allows us to incorporate preliminary information; it is global and not constrained to some local neighbourhood in parameter space and it readily yields information on parameter robustness and confidence. Here we develop the theoretical framework and apply it to a range of exemplary problems that highlight how we can improve experimental investigations into the structure and dynamics of biological systems and their behavior.

## Author Summary

For most biological signalling and regulatory systems we still lack reliable mechanistic models. And where such models exist, e.g. in the form of differential equations, we typically have only rough estimates for the parameters that characterize the biochemical reactions. In order to improve our knowledge of such systems we require better estimates for these parameters and here we show how judicious choice of experiments, based on a combination of simulations and information theoretical analysis, can help us. Our approach builds on the available, frequently rudimentary information, and identifies which experimental set-up provides most additional information about all the parameters, or individual parameters. We will also consider the related but subtly different problem of which experiments need to be performed in order to decrease the uncertainty about the behaviour of the system under altered conditions. We develop the theoretical framework in the necessary detail before illustrating its use and applying it to the repressilator model, the regulation of Hes1 and signal transduction in the Akt pathway.

## Introduction

Mathematical models of biomolecular systems are by design and necessity abstractions of a much more complicated reality [1], [2]. In mathematics, and the theoretical sciences more generally, such abstraction is seen primarily as a virtue which allows us to capture the essential features or defining mechanisms underlying the workings of natural systems and processes. But while qualitative agreement between even very simple models and very complex systems is easily achieved, formally assessing whether a given model is indeed good (or even just useful) is notoriously difficult. These difficulties are exacerbated in no small measure for many of the most important and topical research areas in biology [3]–[5]. The regulatory, metabolic and signalling processes involved in cell-fate and other biological decision-making processes are often only indirectly observable; moreover, when studied in isolation their behavior can often be markedly altered compared to the experimentally more challenging *in vivo* contexts [6]. The so-called “inverse problem” — to learn, construct or infer mathematical or mechanistic models from experimental data — is often considered (see e.g. Brenner [7]) as one of the major problems facing systems biologists.

These challenges have prompted the development of novel statistical and inferential tools, required to construct (or improve) mathematical models of such systems. We can loosely group these methods into (i) those aimed at reconstructing network models [8]–[10] (using correlations or statistical dependencies in observed datasets), (ii) methods to estimate (biochemical reaction) rate parameters of models describing the dynamics of biological systems [11]–[13], and (iii) approaches that allow us to rank or discern between different candidate models/hypotheses [14], [15]. The first set of challenges is typically faced when dealing with new systems where little information is known, and where network-inference algorithms offer a convenient way of generating novel mechanistic hypotheses from data. Here we address the second point. In particular, we start from a model that describes how the abundances of a set of molecular entities, 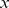, change with time, 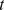; the rate of change in 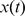 over time is typically described in terms of (ordinary, partial or stochastic) differential equation systems,
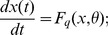
where 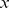 is a 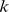-dimensional vector describing the system's state and 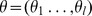 is an 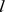-dimensional vector containing the model parameters. Finally, 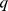 denotes the particular experimental setup under which data are collected. This dependence is generally tacitly ignored but, as we will show below, explicitly incorporating the experimental approach (and the fact that different experimental choices are typically available) into the model and any down-stream statistical analysis allows us to develop strategies that yield more detailed insights into biological systems, and better models thereof. The aims of the present study are to develop experimental design strategies that allow us to infer the (unknown or only poorly known) model parameter, 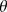, and to reduce the uncertainty in the predicted model behavior.

Inferential tools have been developed that, given some observed biological data and a suitable mathematical candidate model, provide us with parameters that best describe the system's dynamics. Unfortunately obtaining reliable parameter estimates for dynamical systems is plagued with difficulties [16], [17]. Usually sparse and notoriously noisy data are fitted using models with large number of parameters [18]. As a result, over-parameterized models tend to fit to the noisy data but may loose confidence in predictive behavior. Conventional fitting approaches to such data routinely fail to capture this complexity by underestimating the uncertainty in the estimated parameters, which substantially increases the uncertainty in prediction of model behavior.

We use 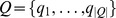 to denote the total set of available experimental assays that could be used to probe a system in a given situation. These might, for example, include knock-out or knock-down mutants, transcriptomic or proteomic assays, different time-courses or different environmental conditions (or both), etc. Here we remain very flexible as to which type of experimental setup is included in 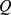, but merely acknowledge that it is rarely possible to probe all important aspects of a system simultaneously. Instead different techniques require different sample preparations etc , and therefore separate experiments. Here we account also for the possibility, that for two different experimental set-ups, 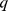 and 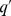, the mathematical model may differ; therefore the dependence on 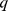 is made explicit in our notation 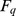. For example, if species 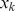 is knocked out in experiment 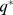, we can ignore any terms referring to it when modelling 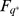.

Performing different experiments is costly, however, in terms of both money and time, and not all experiments are equally informative. Ideally we would like to perform only those experiments which yield *substantial* and *relevant* information. We regard any information that decreases our uncertainty about model parameters or model predictions as *relevant*. As we will show below, what is *substantial* information is then easily and naturally resolved. We will show, for example, that experimental interventions differ in the amount of information they provide e.g. about model parameters. Equally some experiments provide insights that are more useful for making predictions about system behavior than others. It may seem surprising that we consider parameter inference and prediction of output separately, but this merely reflects the fact that not all parameters contribute equally to system output: varying some parameters will have huge impact on the output, while varying other parameters will lead to negligible changes in the output. By making the reduction in uncertainty of predicted model behavior the target of experimental design we explicitly acknowledge this.

Experimental design in systems biology is different from classical experimental design studies. The latter theory was first developed at a time when the number of alternative hypotheses was smaller than the amount of available data and replicates [19]. Systems biology, on the other hand is hypotheses rich and data rarely suffice to decide clearly in favour of one model unambiguously. Moreover for dynamical systems, as a host of recent studies have demonstrated, generally less than half of the parameters are tightly confined by experimental data [16], [17]. Together these two challenges have given rise to a number of approaches aimed at improving our ability to develop mechanistic models of such systems. Here we meld concepts from Bayesian inference and information theory to guide experimental investigations into biological systems to arrive at better parameter estimates and better model predictions.

Several authors have used the information theoretical framework, in particular the expected gain in Shannon information to assess the information content of an experiment [20]–[24]. Although the methodology of Bayesian experimental design is well established, its applicability has been computationally limited to small models involving only several free parameters. Recently their use for systems biology becomes possible as a result of increased computational resources. Vanlier *et. al* proposed an approach that uses the Bayesian predictive distribution to asses the predictive power of experiments [25]. Huan and Marzouk used a framework, which is similar to ours, in that it maximizes mutual information via Monte Carlo approximation to find optimal experiments [26]; but they only focus on parameter inference and ignore prediction. Furthermore they only apply their method to systems with small number of parameters. Here we demonstrate how such an approach can be utilized to analyse multi-parameter models described by ordinary differential equations (ODEs) regarding both, parameter inference and prediction of system behavior. The latter is especially useful when one aims to predict the outcome of an experiment which is too laborious or impossible to perform. Our approach improves on previous methods [25]–[32] in a number of ways: first we are able to incorporate but do not require preliminary experimental data; second, it is a global approach that is not limited to some neighbourhood in parameter space unlike approaches solely based on e.g. the Fisher information [29], [33]; third, we obtain comprehensive statistical predictions (including confidence, sensitivity and robustness assessments if desired); and we are very flexible in the type of information that we seek to optimize.

Below we first develop the theoretical concepts before demonstrating the use (and usefulness) of the Bayesian experimental design approach in the context of a number of biological systems that exemplify the set of problems encountered in practice. In order to demonstrate the practical applicability of our approach we investigate two simple models (repressilator and Hes1 systems), as well as a complex signalling pathway (AKT) with experimentally measured dynamics.

## Results

### Information content of experimental data

To achieve their full functionality mathematical models require parameter values that generally need to be inferred from experimental data. The extraction of this information is, however, a nontrivial task and is further compounded by the need to assess the statistical confidence of parameter estimates. In the Bayesian framework for example, we seek to evaluate the conditional probability distribution, 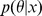, which relates to the prior knowledge 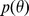 and the distribution of data, 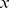, given parameters, 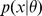, via Bayes' formula
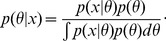
(1)The posterior probability density function, 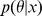, describes the probability of finding a parameter 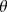 in the volume element 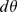 of parameter space, given the data, the model and the prior information. From this distribution we can obtain all relevant information about the parameters: how sensitive the solution is to varying them individually or together; how correlated different parameters are with one another; if there are non-linear dependencies among the parameters; and the level of precision with which each parameter is known in light of the data. A highly readable and enlightening account of the Bayesian formalism is given e.g. in [34]. Finding the posterior, 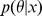, is usually achieved by means of powerful (if costly) computational algorithms such as Markov chain Monte Carlo (MCMC) and sequential Monte Carlo (SMC) methods, which also exist in the approximate Bayesian computation (ABC) framework [15], [35], [36].

**Figure 1:**
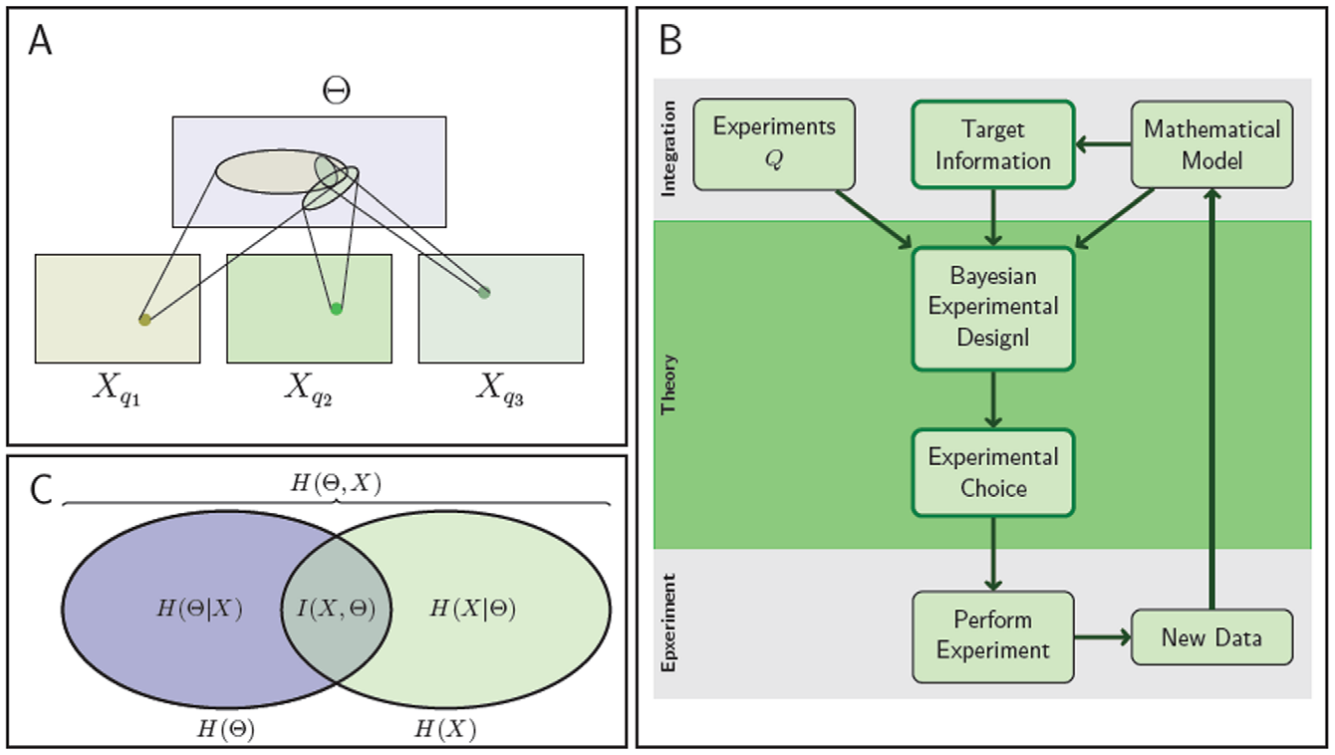
Information content of experimental data and flow chart of the experimental design method. (A) The regions of plausible parameters values for three different experiments. Each ellipse defines the set of parameters which are commensurate with the output 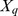 of an experiment 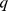. In this example, the data 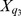 leads to the most precise inference of the parameters. The parameters which explain the output of all the three experiments are at the intersection of the three ellipsoids. (B) Flowchart of the experimental design method. Given a mathematical model of the biological system, a set of experiments and the target information —which can be either a set of parameters to infer or a description of the experiment to predict — the Bayesian Experimental Design method determine the experiment to carry out. Once the experiment has been performed, the experimental data are then used to provide target information and to improve the model. Thereafter, the process can be iterated to select other experiments in order to improve the accuracy of the target information. (C) Link between the total and conditional entropies and the mutual information of experimental data 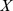 and parameters 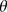.

Rather than providing a single parameter estimate the posterior distribution allows us to assess how well a parameter is constrained by data (see Figure 1 A). More formally, we measure the uncertainty about a parameter information-theoretically in terms of the Shannon entropy [37],
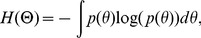
(2)for the prior and
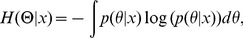
(3)for the posterior. The information gained by collecting data 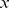 can then be expressed as 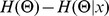. The output of the experiment, however, is in turn “random” with distribution 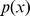, and therefore the average posterior uncertainty is
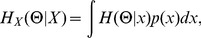
(4)which leads to the average information gain called mutual information between 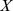 and 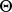,
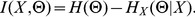
(5)When faced with different experimental setups, 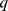, and hence different datasets, 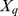, choosing the set(s) which maximize 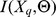 will provide the best insights into the system via improved parameter estimates [20], [38], [39]. This observation is the basis of our experimental design methodology which consists of computing the mutual information 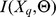 for every experiment 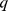 and selecting the experiment resulting in the highest mutual information (see *Methods* for computational details). Once the chosen experiment has been carried out, the new data are used to update the model and the posterior distribution of the parameters (see Figure 1 B).

Given the importance of the predictive role of mathematical modelling it is also of interest to reduce the uncertainty of model predictions; intriguingly and perhaps counterintuitively — but demonstrably and provably (see below and *Supplementary Material*) — better parameter estimates are not necessarily required for better, more certain model predictions. Therefore, instead of focussing on parameter inference we can directly seek to identify the experimental condition 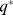 which minimizes the uncertainty in the predicted trajectories 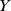. Analogously to the previous case minimizing uncertainty in predictions of 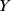 means to maximize mutual information between 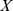 and 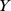 (see *Methods*):
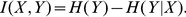
(6)


Below we use three examples of different complexity to show how this combination of rigorous Bayesian and information theoretical frameworks allows us to design/choose optimal experimental setups for parameter/model inference and prediction, respectively.

### Experiment selection for parameter inference

**Figure 2:**
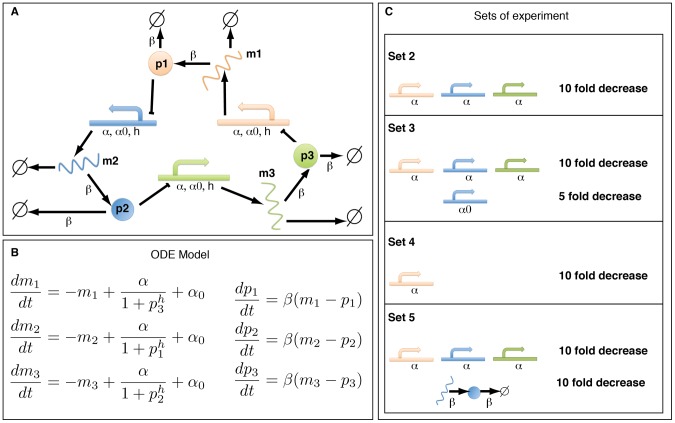
The repressilator model (as described in [40]) and the set of possible experiments. (A) Illustration of the original repressilator model. The model consists of 3 mRNA species (coloured wavy lines, labeled 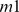, 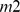 and 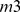) and their corresponding proteins (circles shown in the same color with labels 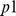, 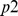 and 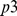). The 3 regulatory DNA regions are not modeled explicitly but included in the mRNA production process. They are shown for illustration purpose only. (B) The ordinary differential equations which describe the evolution of the concentration of the mRNAs and proteins over time. (C) Four potential modifications of the wild-type model. For each experimental intervention the modified parameters are listed (colours are as in A). The modifications of the wild-type model consist of decreasing one or several of the parameters of the system: in sets 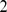, 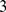 and 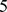, the regime of the parameter 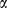 is changed; in sets 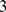, 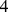 and 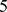, respectively, parameters 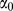, 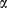 and 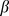 are modified for only one gene which breaks the symmetry of the system.

To investigate the potential of our experimental design method for parameter estimation we first apply it to the repressilator model, a popular toy model for gene regulatory systems [40]. It consists of three genes connected in a feedback loop, where each gene transcribes the repressor protein for the next gene in the loop (see Figure 2 A and B).

To infer the parameters of this model, 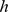, 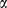, 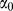, and 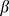, we propose 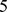 sets of possible experiments: the original repressilator model (set 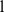) which is described in Figure 2 A and corresponds to the ordinary differential equations in Figure 2 B, and 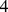 modifications of the original model, see Figure 2 C. The suggested 4 experiments are hypothetical, but can be linked to potential experiments. For example, a decrease in the parameter 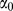 corresponds to a decrease of the basal transcription rate, which could be achieved with inhibitors or site-directed mutation of the corresponding transcription factor binding site. The proposed modifications can lead to different dynamics, and this in turn can lead to a higher mutual information between the parameters and the resulting mRNA and protein trajectories, which are here the output of the system. The information content increases as differences in the outputs resulting from different parameter values increase. In Figure S1, we illustrate the link between the increase in mutual information and the dynamics of the system for three different regimes.

**Figure 3:**
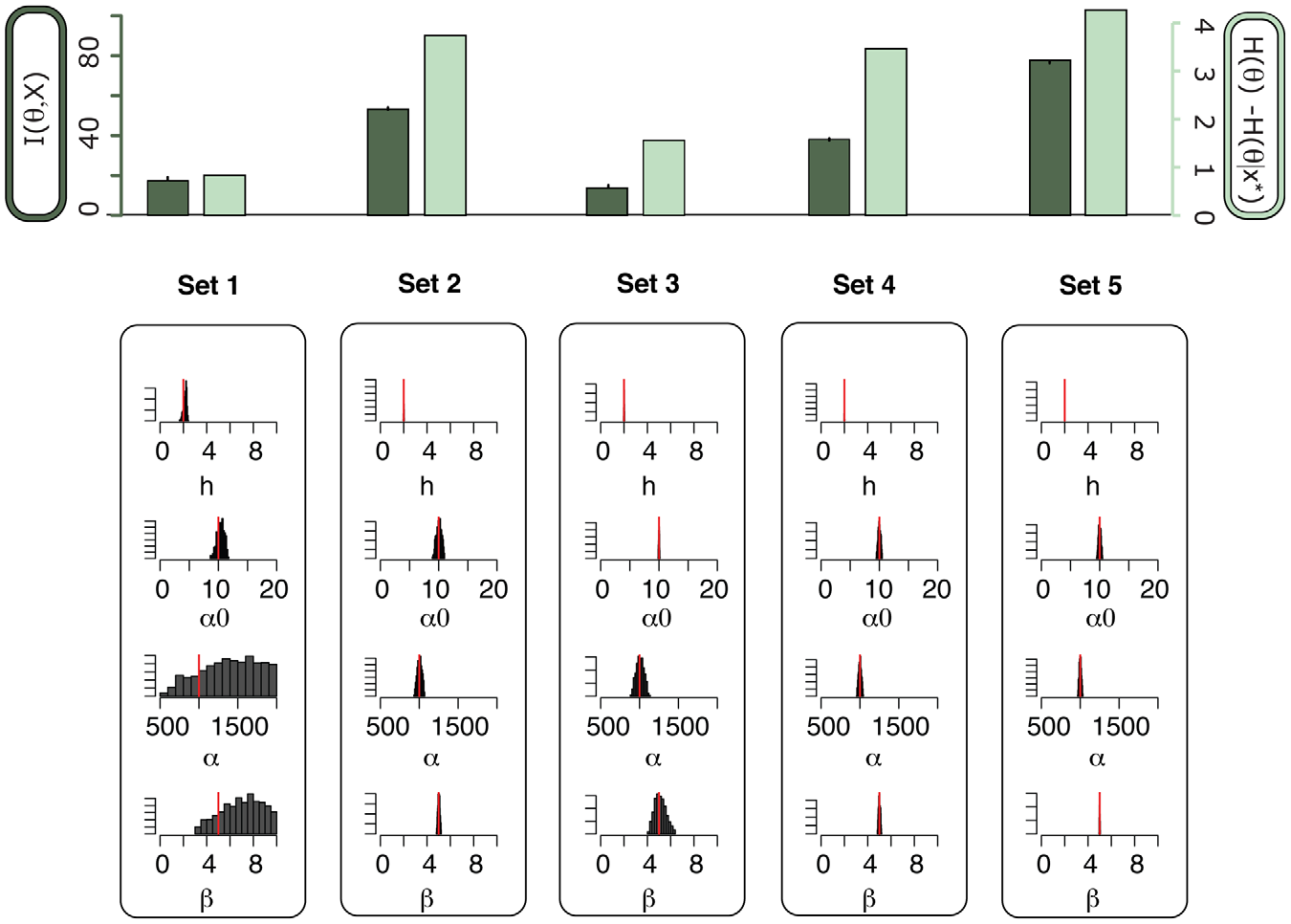
Experiment choice for parameter inference in the repressilator model. Top: The mutual information 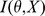 between the parameters 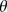 and the output of each set of experiment (in dark green), and the entropy difference between the prior distribution and the posterior distribution. The posterior distribution is based on data obtained from simulation of the system for each experiment (in light green). The error bars on the mutual information barplots show the variance of the mutual information estimations over 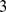 independent simulations. Bottom: For each set of experiment we show the histogram of the marginal of the posterior distribution of every parameters. The red line indicates the true parameter value.

To determine which experiment to carry out we compute the mutual information between the parameter prior distribution and the system output via Monte-Carlo estimation. We use uniform priors over 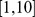 for 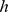, over 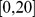 for 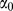, over 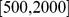 for 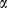, and over 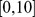 for 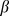. Figure 3 shows that experiment 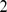 and 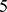 have highest mutual information, i.e. carrying out those experiments will decrease the uncertainty in the parameter estimates most. To confirm this we simulate data for the 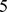 experiments using the parameter 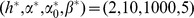. The simulated data are shown in Figure S2. Based on these data we perform parameter inference using an approximate Bayesian computation approach [41] for each experiment separately and compare the posterior distributions shown in Figure 3. We observe that using the data generated from set 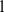 (original repressilator system) only 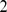 parameters can be inferred with confidence: 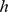 and 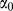. By contrast, the data generated by set 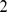 and set 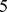 allow us to estimate all 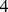 parameters. In addition, for each experiment we compute the reduction of uncertainty from the prior to the posterior distribution. The results are consistent with the results using mutual information and confirm that we should choose experiment 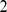 or 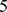 for parameter inference. In practice not all molecular species may be experimentally accessible and it is therefore also of interest to decide which species carries most information about the parameters. We can estimate the mutual information between the parameter and each species independently, and, for example, for experimental set 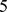 we observe that mRNA 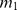 and 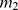 as well as protein 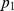 carry equally high information. This mutual information for each mRNA and each protein is plotted in Figure S3.

**Figure 4:**
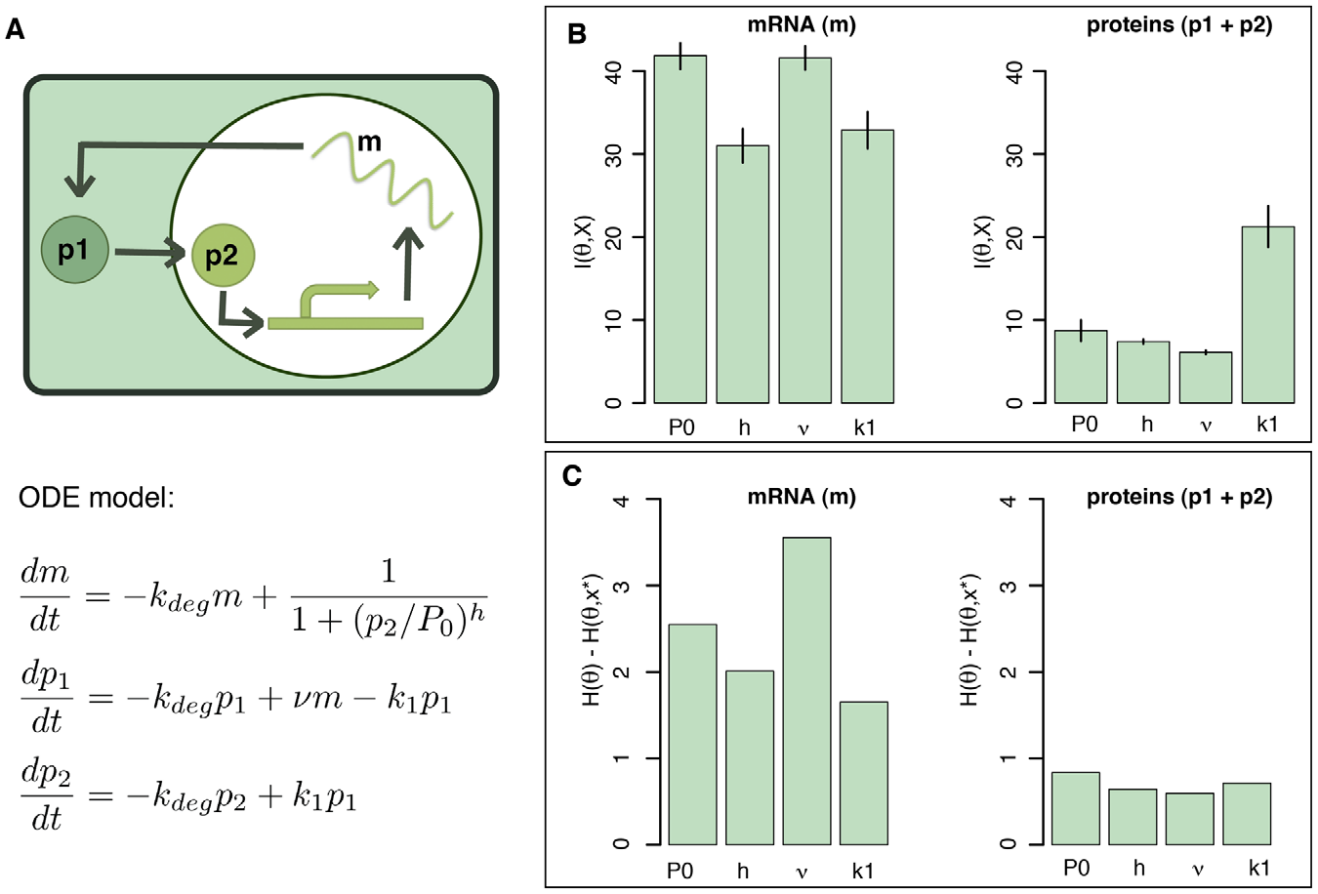
Experiment selection for parameter inference in the Hes 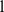 model. (A) Diagram of the Hes 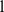 model and the associated ordinary differential equations. The model shows a cell (green box) with its nucleus (white circle). Hes 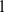 mRNA (wavy line with label 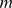) is produced inside the nucleus and translated into Hes 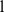 cellular protein (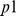), which in turn is then transported into the nucleus and becomes nuclear Hes 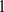 protein (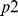). 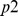 is regulating the production of 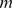. (B) The mutual information between each parameters and the output of the system for respectively mRNA measurement (left) and total cellular Hes 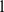 measurement (right). The barplot represents the mean and the variance over 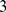 repetitions of the Monte-Carlo estimation. (C) Estimation of the difference between the entropy of the parameter prior and the entropy of the posterior distribution given one dataset. The dataset results from simulation of the Hes 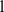 system. The parameter 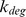 was set to be 0.03 min^−1^ as experimentally determined by [42].

Sometimes we are interested in estimating only some of the parameters, e.g. those that have a direct physiological meaning or are under experimental control. To investigate this aspect we consider the Hes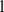 transcription factor that plays a number of important roles, including in the cell differentiation and segmentation of vertebrate embryos. Oscillations observed in the Hes1 system [42] might be connected with formation of spatial patterns during development. The Hes1 oscillator can be modelled by a simple three-component ODE model [43] as shown in Figure 4 A. This model contains 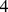 parameters, 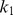, 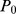, 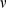, and 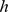, and 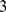 species: Hes 1 mRNA, 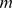, Hes 1 nuclear protein, 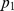, and Hes 1 cytosolic protein, 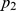. It is possible to measure either the mRNA (using real-time PCR) or the total cellular Hes 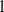 protein concentration 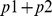 (using Western blots). We investigate whether protein or mRNA measurements provide more information about the model parameters. Thus we estimate the mutual information between mRNA and parameters, and between protein and parameters. Figure 4 B shows that mRNA measurements carry more information about all of the parameters.

This can again be further substantiated by simulations. We perform parameter inference based on such simulated data (simulated data are shown in Figure S5) and compute the difference between the entropy of the prior and that of the resulting posterior distribution. The results shown in Figure 4 C are consistent with the predictions based on mutual information: mRNA measurements carry more information for parameter inference. Interestingly, however, although the mutual information computation indicates that the protein measurements should contain more information about parameter 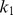 than about the other parameters, this is not confirmed by the difference in entropy result for this simulated data set. This divergence is due to the fact that the mutual information measures the amount of information contained *on average* over all the possible behaviours of the system, whereas Figure 4 C represents the decrease in entropy from the prior to the posterior distribution given *specific data*. The differences in entropy for other data sets simulated using different parameter regimes are thus in better agreement with the mutual information results. We confirm this in Figure S6 where we show the results of the same analysis based on a different simulated data set.

### Experiment selection for prediction

**Figure 5:**
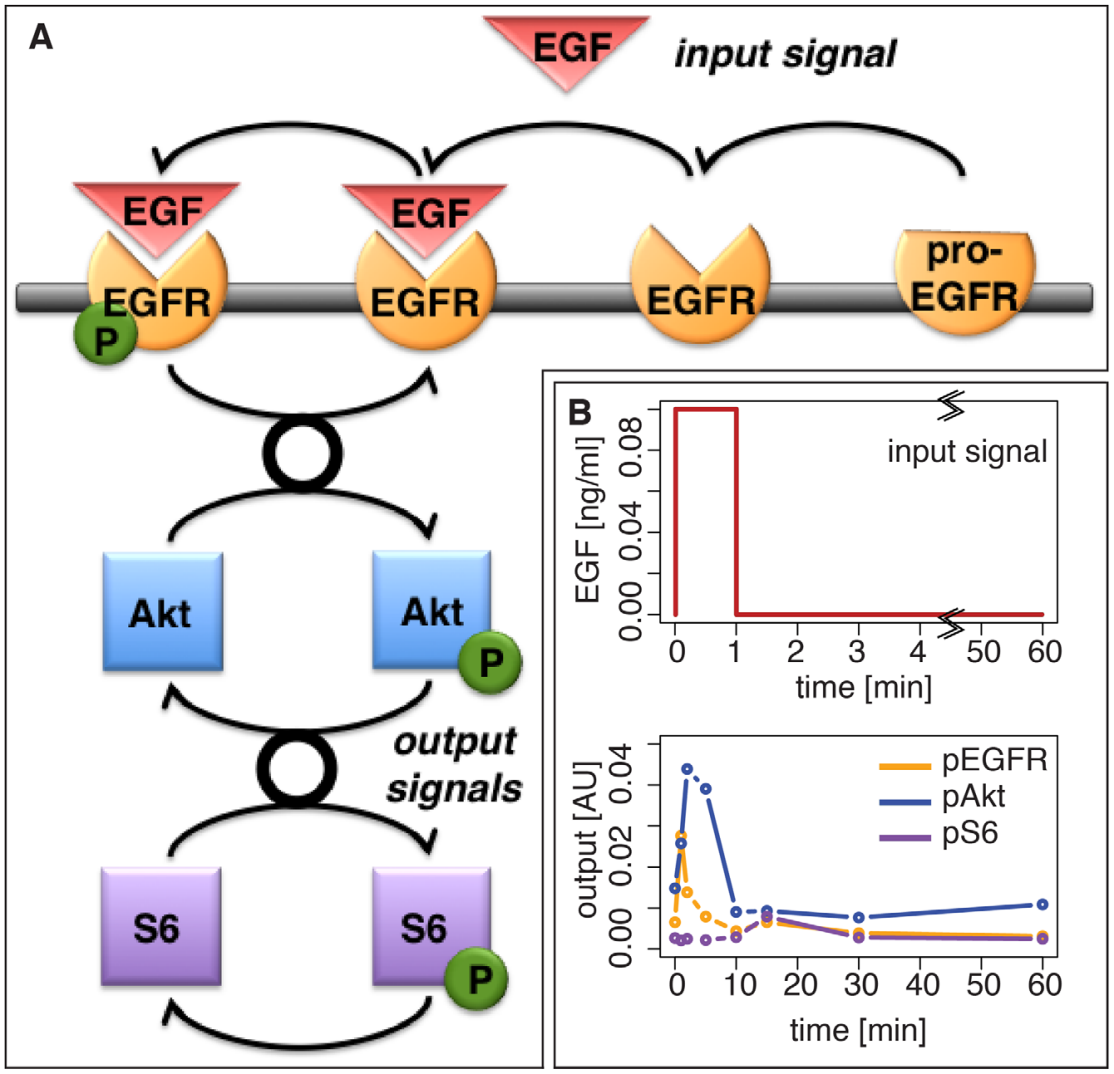
The EGF-dependent AKT pathway and an initial dataset. (A) Diagram of the model of the EGF-dependent AKT pathway. Epidermal growth factor (EGF, red triangle) is a stimulus for a signalling cascade, which results in the phosphorylation (green circle) of Akt (blue square) and S6 (purple square). EGF binds to the EGF membrane receptor EGFR (orange), which is generated from a pro-EGFR. The Binding results in the phosphorylation of the receptor, which consequently leads to the activation of downstream cascades (thick black circle). This simplified model was shown to capture the experimentally determined dynamics [45]. (B) A impulse input of EGF over 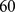 seconds with an intensity of 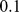 ng/ml (top) and the resulting time course of phosphorylated EGF receptor (pEGFR), phosphorylated Akt (pAKT) and phosphorylated S6 (pS6) in response to this stimulus (bottom). Data were provided by the authors of [45].

We next focus on a scenario where we aim to predict the behaviour of a biological system [44] under conditions for which it is not possible to obtain direct measurements. We consider as an example the phosphorylation of Akt and ribosomal binding protein S6 in response to a epidermal growth factor (EGF) signal. Figure 5 A shows the pathway of interest: the EGF growth factor binding to the activated receptor EGFR leads to phosphorylation of EGFR and a signal cascade which results in the phosphorylation of Akt (pAkt) which in turn can activate downstream signalling cascades and leads to the phosphorylation of S6 (pS6); a corresponding mathematical model is shown in Figure S7 [45].

**Figure 6:**
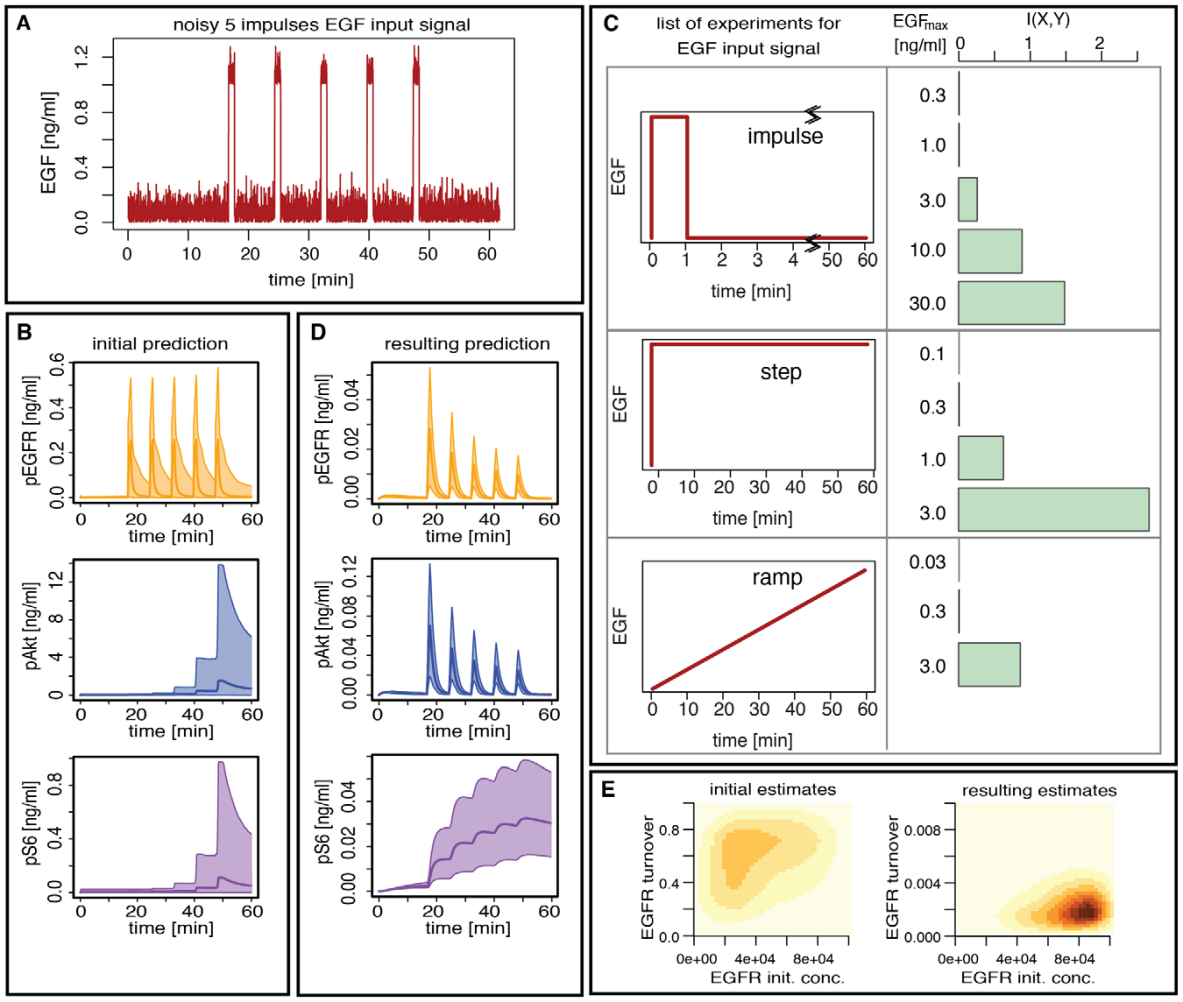
Experiment selection for prediction in the EGF-dependent AKT pathway. (A) The noisy 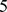-impulses EGF input signal: the 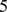 pulses are of intensity 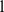 ng/ml and length 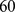 seconds spaced by 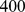 seconds with an additive background noise which is the absolute value of a gaussian white noise of variance 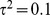. (B) The predicted time course of the proteins pEGFR, pAKT and pS6 under the noisy 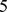-impulses EGF input signal based on the initial dataset. (C) The mutual information between the time course of the 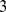 species of interest under the noisy 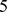-impulses EGF input signal and the time course of the species under each of the following 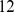 possible experiments: an impulse stimulus of length 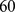 seconds with 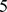 possible intensity (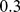, 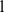, 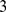, 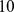 and 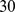 ng/ml), a step stimulus of length 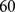 minutes with 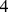 possible intensity (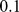, 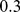, 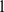 and 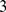 ng/ml) and a ramp stimulus of length 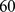 minutes with 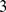 possible final intensity (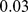, 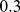 and 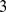 ng/ml). (D) The predicted time course of the proteins pEGFR, pAKT and pS6 under the noisy 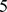-impulses EGF input signal based on the outcome of the step stimulus with intensity 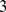 ng/ml, which is the experiment with the highest mutual information. The scale of the y-axis is different for Figures (B) and (D). (E) The posterior distribution of two parameters (EGFR turnover and EGFR initial condition) when using the initial dataset alone (left) and when using the initial dataset and the outcome of the step stimulus with intensity 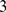 ng/ml (right). The scale of the EGFR turnover is the prior range for the figure on the left panel whereas it is 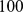 times smaller for the figure in the right panel.

We are interested in predicting the dynamics under multiple pulsed stimuli with EGF in the presence of background noise, as shown in Figure 6 A. We consider 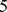 pulses of intensity 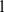 ng/ml and length 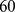 seconds spaced by 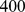 seconds with additive background noise. This input is difficult to realize in an experimental system (let alone an animal or clinical setting). Using an initial data set, see Figure 5 B, we can infer system parameters using ABC SMC. The resulting fit to the data using the inferred parameters are shown in Figure S8. From the resulting posterior distribution we then sample 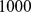 parameter combinations and simulate the model with the 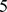-pulse-stimulus in order to predict the time courses of phosphorylated EGF receptor (EGFR), phosphorylated Akt and phosphorylated S6; based on just the estimated parameters these predictions are, however, associated with high uncertainty, see Figure 6 B.

To obtain better predictions we can use data from other experiments measuring the time course of the 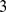 species of interest for a experimentally more straightforward input signals chosen from among 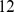 possible stimuli: impulse, step or ramp stimuli with different EGF concentrations (see Figure 6). To determine which of those inputs would result in the most reliable predictions we compute the mutual information between the time courses for the different potential experimental inputs and the time-course of the target 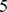-impulse noisy stimulus. We incorporate initial information about model parameters by computing the mutual information based on the posterior distribution inferred above. Figure 6 C shows that a step stimulus of intensity 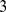 ng/ml has the highest mutual information and therefore reduces the uncertainty of the predicted behavior of our target stimulus pattern.

In Figure 6 D we show that this does indeed yield much improved predictions compared to the initial prediction. This reduction in uncertainty about predicted model behaviour results from the difference in the posterior distributions obtained under different stimulus regimes; by focussing on predictive ability we focus implicitly on data that is informative about those parameters that will affect the system behaviour most under the target (5-pulse) stimulus. The posterior distributions are represented in Figure 6 E for two parameters, the EGFR turn over and the EGFR initial concentration, which appear to be essential for the prediction of the evolution of Akt/S6 phosphorylation patterns under the 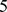-pulse stimulus. Those two parameters were not inferred using the initial dataset alone, whereas the addition of the outcome of the step stimulus experiment suggested by our methods infers these parameters at the required precision.

This ability to predict the time courses extends to much greater signal distortion and even with a noise level of 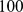 percent of the signal intensity we find that our experimental design method yields similar improvements in the predictions. This additional analysis of the new input signal is shown in Figure S10 and S11. We observe that the direct target (EGFR receptor) as well as activated AKT (pAKT) efficiently filter out the noise but capture the 5 pulses; EGFR activation quickly returns to base level in response to the higher frequency background noise. This indicates that there might be a constant low concentration of activated EGFR (pEGFR), but the activation of S6 has very different characteristics and is far less robust to noise. The level of noise is amplified as can be observed in the pS6 time course. This might suggest that the downstream molecule pS6 has a longer time delay to react to a signal. Moreover, pS6 does not have time to relax to its baseline between the 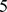 pulses, which leads to incremental signal amplification. This behavior fits with the low-pass filter characteristics previously described [45]. In further support earlier studies [46] found that a downstream molecule can be more sensitive to an upstream activator than the direct target molecule of the activator. This might explain that the activation of EGFR and AKT is more robust to noise than the downstream molecule, S6.

## Discussion

We have found that maximizing the mutual information between our target information — here either model parameter values or predictions of system behaviour — and the (simulated) output of potentially available experiments offers a means of arriving at optimally informative experiments. The experiments that are chosen from a set of candidates are always those that add most to existing knowledge: they are, in fact, the experiments that most challenge our current understanding of a system.

This framework has a number of advantages: First, we can simulate cheaply any experimental set-up that can in principle be implemented; second, using simulations allows us to propagate the model dynamics and to quantify rigorously the amount of (relevant) information that is generated by any given experimental design; third, our information measure gives us a means of meaningfully comparing different designs; finally, our approach can be used to design experiments sequentially — our preferred route as this will enable us to update iteratively our knowledge of a system along the way — or in parallel, i.e. selecting more than one experiment. Previous approaches had taken a more local approach [25], [29], [30], [47] that relied on initial parameter guesses and often data; our approach also readily incorporates different stimulus patterns [48].

Here we have focussed on designing experiments that increase our ability to estimate model parameters and to predict model behaviour. The latter depends on model parameters in a very subtle way: not all parameters affect system output equally and under all conditions. Target conditions could, for example, include clinical settings which are generally not experimentally amenable (at least in early stage research); here the current approach offers a rationale for designing [49] therapeutic interventions into complex systems based on investigations of suitable model systems. In this study we provide an approach to chose the optimal experiment out of a finite and discrete set of possible experiments. Experimental design with a continuous set of experiments requires a different approach, as for example shown in [50].

With an optimal design we can overcome the problems of sloppy parameters [16] (which are, of course, dependent on the experimental intervention chosen [17]) and can narrow down the posterior probability intervals of parameters. We would like to reiterate, however, the importance of considering joint distributions rather than merely the marginal probabilities of (or the confidence regions associated with) individual parameters: parameters of dynamical systems tend to show high levels of correlation (i.e. we can vary them simultaneously in a way that does not affect the output of the system — at least in some areas of parameter space) and their posterior probability distributions often deviate from normality (which also motivated the use of information theoretical measures which can deal with non-linearities and non-Gaussian probability distributions).

We tested and applied our approach in three different contexts: while the repressilator serves as a toy model with hypothetical data and experiments, the Hes1 transcription regulatory system and the EGF induced Akt pathway are relevant biological systems. The question to answer in the Hes 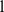 system, which molecular species to measure, is a common question in laboratories. The presented study of the Akt system does not only demonstrate how to chose experiments for prediction of system behaviours, but it is also an example for stimulus design.

The approach we presented here yields the potential for model discrimination or checking the target of our analysis [48], [51], and, for example, choose experimental designs that maximize our ability to distinguish between competing alternative hypotheses or models. All of this is straightforwardly reconciled in the Bayesian framework, which also naturally lends itself to such iterative procedures where “today's posterior” is “tomorrow's prior” and models are understood increasingly better as new, more informative data are systematically being generated.

## Methods

### Information theoretic design criteria

Our aim is to choose an experiment 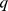 from a set of candidate experimental setups, 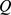, which either reduces uncertainty about model parameters or uncertainty of an outcome of a particular condition 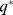 for which data are impossible or difficult to obtain. In the information theory framework, these two goals boil down to determining an experiment 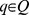 which contains maximal information about the parameter or the desired predictions for condition 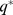. In order to present in more details these two goals and for better understanding we revise some concepts of information theory [34]. We define the entropy 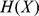 of a random variable 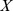, which measures the uncertainty of the random variable,
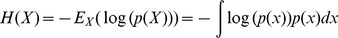
(7)and the mutual information 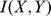 between two random variables 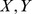, which is the reduction of the uncertainty that knowing 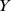 provides about 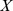,
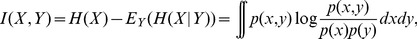
(8)where 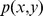 is the joint probability density function of 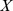 and 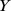 while 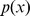 and 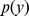 are the marginal probability density functions. We denote by 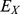 the expectation with respect to the probability distribution of 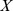. Here we follow the convention where capital letters stand for random variables while lower-case stand for a particular realization of a random variable.

### Reducing uncertainty in model parameters

We first consider the task of choosing an experiment that will on average provide most information about model parameters measured through the reduction in their respective uncertainties. In the information theoretic language, as by Lindley [20] and later by Sebastiani and Wynn [52] the initial (prior) uncertainty is given by the entropy 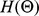 of the prior distribution 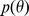, which after data 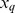 have been collected (in experimental setup 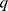) gives rise to the entropy 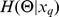 of the posterior distribution 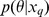. The information gained about the parameter by collecting the data 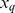 is then 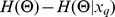. On average, however, the decrease of uncertainty about 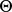 after data are collected in an experiment 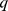 is given by 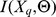. Therefore, in order to reduce parameters' uncertainties one should choose an experiment that maximizes the mutual information between 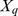 and 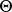.

Here we specifically consider models such that the output is of the form
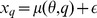
(9)where 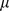 is a deterministic function and 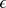 an uncorrelated, zero mean, gaussian random variable with variance 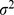 (our approach is readily extended to stochastic systems). In such a model maximization of mutual information 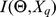 is equivalent to maximization of the entropy 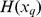. This observation described first in [52] results directly from the fact that the mutual information 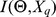 can be written as the difference between 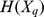 and 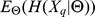 and that
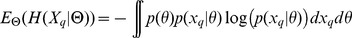
does not depend on the experiment 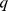. Indeed, Equation (9) implies that 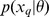 is the probability of the experimental noise 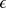. Therefore maximization of 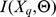 is equivalent to maximization of 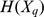. However, this is only the case for the mutual information between the ouput 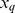 of an experiment 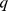 and the parameter of the system 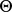. Whenever we are interested in the increase of information about only one component of the parameter vector, or in reducing uncertainty about an experimental outcome we need to use the mutual information and not the entropy.

### Reducing uncertainty in an experimental outcome

Similar reasoning leads us to a criterion for selecting an experiment 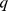 that reduces uncertainty about predictions for the system output under a different set of conditions or experiment 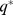. Choosing 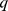 that maximizes 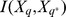 leads to an experiment that on average reduces the uncertainty of predictions for condition 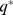 most. This can be seen by rewriting (8)
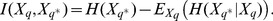
(10)


### Estimation of the mutual information

The mutual information for models of type (9) can be estimated using Monte Carlo simulations [26], [53]. We first focus on the mutual information between parameters 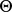 and the output 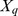 of an experiment 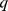, which can be written as a function of the prior distribution 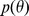, the probability of the output given the parameter 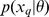 and the evidence 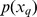 as follows
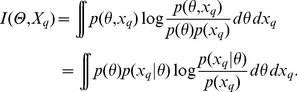
(11)Drawing a sample 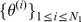 from the prior distribution 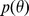 we obtain a Monte-Carlo estimate,
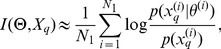
(12)where for all 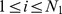, 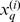 is an output of the system for the parameter 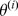. For models of type (9) 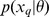 is the probability density function of a Gaussian distribution with mean 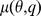 and covariance 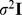 taken at 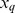. To compute the quantity in (12) we have to estimate the evidence 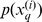, which can be done via Monte Carlo simulation: given a 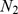-sample 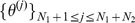 drawn independently from the prior distribution 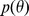 with 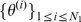 we have
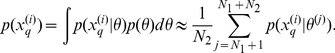
(13)Combining equations (12) and (13), we obtain the following estimate of the mutual information between the parameter 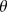 and the output 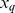,
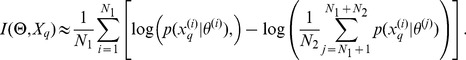
(14)


Similarly, we can estimate the mutual information between any single component 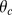 of the 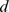-dimensional parameter vector, 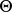, and the output 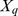. We denote by 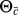 the 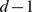-dimensional vector containing all the components of 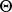 except the 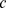-th one. Integrating over 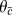, the mutual information between 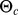 and 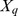 is equal to
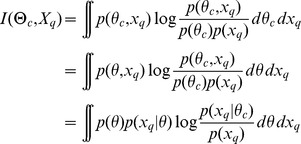
and can be estimated through Monte Carlo simulation by
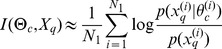
(15)As previously the evidence 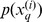 can be estimated from a sample drawn from the prior without great difficulty. On the other hand, the estimation of the numerator 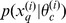 requires an additional integration over 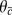. We then have
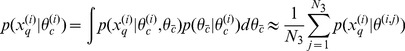
(16)where for each 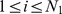, 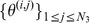 is a sample drawn from 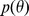 under the constraint that 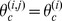. Putting all the terms together, we obtain the following estimation of the mutual information between 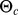 and 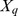: given a sample 
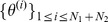
 drawn from 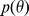 and a 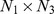-sample 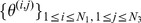 such that for all 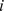, 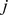, 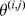 is drawn from 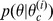,
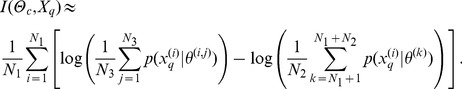
(17)


To finish we consider the estimation of the mutual information between the output of the system for two different experiments 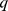 and 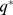. We have
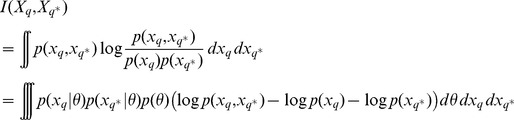
where we used the fact that 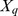 and 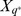 are independent conditionally to a parameter 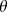. This equation leads to a Monte Carlo estimation from a 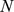-sample 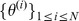 drawn from the prior given by
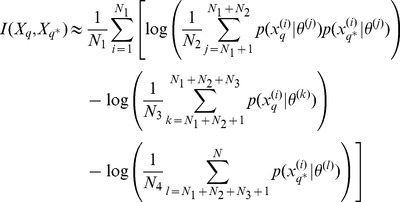
where 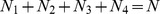.

#### Technical details

We implemented the algorithms in 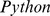. The numerical solutions of the models were obtained using the solver for ordinary differential equations of the package 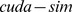 [54], which allows for parallel implementation on graphical processing units (GPUs). The algorithm to obtain the Monte Carlo estimates was also parallelized using GPUs. For the repressilator, we assume measurement noise 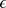 with variance 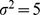. To estimate the mutual information between the output and the parameter, we use 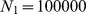 and 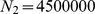. The mutual information between the output of the system and each parameter in the Hes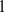 example is computed using 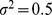, and 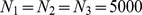. For the AKT model, the variance of the measurement noise is equal to 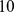, 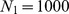, and 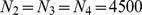.

### Approximate Bayesian Computation (ABC)

Once data 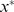 have been collected, we use an Approximate Bayesian Computation framework [55], [56] to infer the posterior parameter distribution 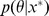. This is a simulation-based method which mainly consists in sampling the parameter space from a prior distribution 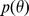, simulating the system for each sampled parameter (often called particle) and selecting the particles such that the simulated data are less than some maximal distance away from the observed data. Those particles define an estimate of the posterior distribution given the observed data:
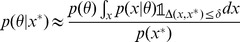
Specifically we use an ABC scheme based on sequential Monte Carlo, which has been developed for likelihood-free parameter inference in deterministic and stochastic systems [41]. We use the implementation of this method of the package called 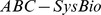 [57].

### Estimation of the entropy

The estimation of entropy has been performed only to test and confirm our experimental choice, which is based on Monte Carlo estimation of mutual information. For each experiment 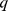 we compute the difference between the entropy 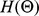 of the prior distribution 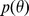 and the entropy 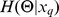 of the posterior distribution 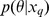. The entropies 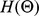 and 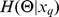 are approximated using a histogram-based estimators. This discretization of the parameter space leads to a change of scale in the entropy measure. This explains why the scales of the differences between estimated entropies and the estimated mutual information differ despite the fact that the mutual information 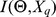 is the expectation over all possible data 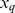 of the difference between 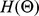 and 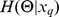. It is also well known that such histogram approach leads to a biased estimate of the entropy [58]. However, since the bias only depends on the number of bins and the sample size, we can compare the estimation results among experiments as long as these algorithm parameters are kept the same.

#### Technical details

To compute the entropy 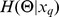 for each experiment 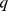 in the repressilator example we compute a 4-dimensional histogram to discretise the posterior distribution (for all 4 model parameters) using 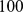 bins for each dimension resulting in a total of 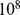 bins. We use the 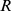 package 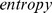 [59] to estimate the entropy. For the Hes 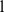 model we computed histograms over the marginals posterior distribution, to measure the entropy of each parameter separately. Here we used 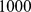 bins.

### Experimental data

The experimental data sets used to investigate the Akt model were collected and published by the lab of S. Kuroda. The data are normalised Western blot measurements as described in [45].

## Supporting Information

Figure S1Information content of different parameter regimes. The mutual information 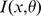 depends on the dynamics of the system given the prior of the system parameters: the more the dynamics for different parameter values differ from each other, the higher is the information content. To visualize this we compute the mutual information between one parameter and the outcome of the system for three different regimes in the repressilator example. Noting that 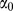 is a bifurcation parameter and 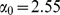 is a Hopf bifurcation point, we choose 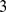 different prior regimes for 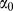: 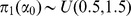, 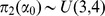 and 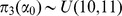. We keep the remaining 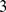 parameters constant: 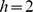, 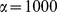 and 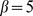. For these three priors we estimate the mutual information 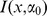 and represent the dynamics of the output of the system. We observe that the dynamics resulting from the first prior regime are most diverse and therefore 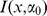 has the highest value (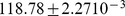) compared to the remaining two parameter regimes (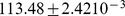 for 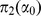 and 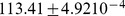 for 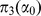). Shown is the bifurcation diagram for parameter 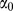 with its stable (solid lines) and unstable (dashed lines) states. Estimation of mutual information was performed for 3 different parameter regimes. For illustration we plot the mean (dark blue), 25 and 75 percentiles (blue) and the 5 and 95 percentiles (light blue) of trajectories simulated with 10000 parameter sets, where 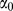 is uniformly sampled and the remaining parameters are kept constant (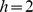, 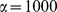 and 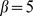).(TIFF)

Figure S2The simulated evolution of the mRNA and protein concentration in the repressilator model for each experimental setup. The parameter vector used for simulations is 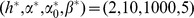. The colours correspond to those in Figure 2. The dots represent the simulated data and the lines correspond to the mean of the species for 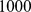 parameters sampled from the posterior distribution computed using ABC SMC.(TIFF)

Figure S3Mutual information between the parameter and each species (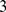 mRNA and 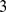 protein measurements) for experiment 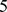 in the repressilator model.(TIFF)

Figure S4The posterior distribution given the data represented in Figure S2. Each subfigure (A to E) corresponds to an experiment (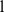 to 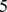). In each subfigure, the diagonal represents the marginal posterior distribution for each parameter and the off-diagonal elements show the correlations between pairs of parameters.(TIFF)

Figure S5Simulated trajectories of the mRNA and protein concentrations (dots). The parameter used for simulation is 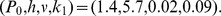 The lines represent the 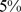 and 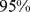 percentiles of the species abundances for 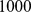 parameters sampled from the posterior distribution computed using ABC SMC.(TIFF)

Figure S6(A) Simulated trajectories of the mRNA and protein concentration (dots) for the parameter 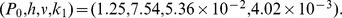 The lines represent the 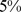 and 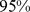 percentiles of the species abundances for 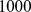 parameters sampled from the posterior distribution computed using ABC SMC. (B) Estimates of the differences between the entropies of the prior and posteriors.(TIFF)

Figure S7Ordinary differential equations which describe the dynamics of the 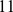 species of the AKT model. The model contains 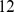 parameters denoted 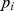, 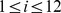. The concentration of the following species (in this order) are denoted by 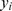, 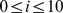: EGF, EGFR, pEGFR, pEGFR-AKT, AKT, pAKT, S6, pAKT-S6, pS6, pro-EGFR and EGF-EGFR.(TIFF)

Figure S8The time course of phosphorylated EGF receptor (pEGFR), phosphorylated Akt (pAKT) and phosphorylated S6 (pS6) in response to an impulse input of EGF over 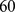 seconds with an intensity of 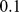 ng/ml (dots). Data are Western blots measurements, described in [45]. The lines represent the 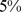 and 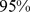 percentiles of the evolution of the species for 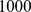 parameters sampled from the posterior distribution computed using ABC SMC.(TIFF)

Figure S9The time course of phosphorylated EGF receptor (pEGFR), phosphorylated Akt (pAKT) and phosphorylated S6 (pS6) in response to a step input of EGF over 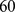 minutes with an intensity of 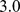 ng/ml (dots). Data are Western blots measurements, which have been generated and published by [45]. The lines represent the 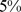 and 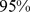 percentiles of the evolution of the species for 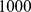 parameters sampled from the posterior distribution computed using ABC SMC.(TIFF)

Figure S10(A) A noisy 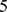-impulses EGF input signal: the 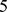 pulses are of intensity 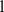 ng/ml and length 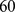 seconds spaced by 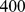 seconds with an additive background noise which is the absolute value of a gaussian white noise of variance 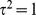. (B) The mutual information between the time course of the 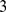 species of interest under the noisy input signal represented in (A) and the time course of the species under each of the following 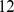 possible experiments: an impulse stimulus of length 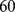 seconds with 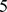 possible intensity (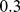, 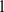, 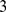, 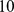 and 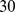 ng/ml), a step stimulus of length 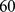 minutes with 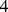 possible intensity (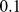, 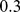, 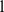 and 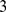 ng/ml) and a ramp stimulus of length 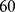 minutes with 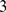 possible final intensity (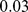, 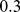 and 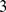 ng/ml).(TIFF)

Figure S11The predicted time course of the proteins pEGFR, pAKT and pS6 under the noisy 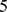-impulses EGF input signal with a noise of high intensity represented Figure S10 A. In the left panel, the prediction is based on the initial dataset whereas in the right panel in addition to the initial data it is also based on the outcome of the step stimulus with intensity 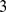 ng/ml, which is the experiment with the highest mutual information. The scale of the y-axis is different for each figure.(TIFF)
