# Supplementary figures and images for: Maximizing the Information Content of Experiments in Systems Biology
Source: PLoS Comput Biol. 2013 Jan 31;9(1):e1002888. doi: 10.1371/journal.pcbi.1002888 (PMC3561087; doi:10.1371/journal.pcbi.1002888)

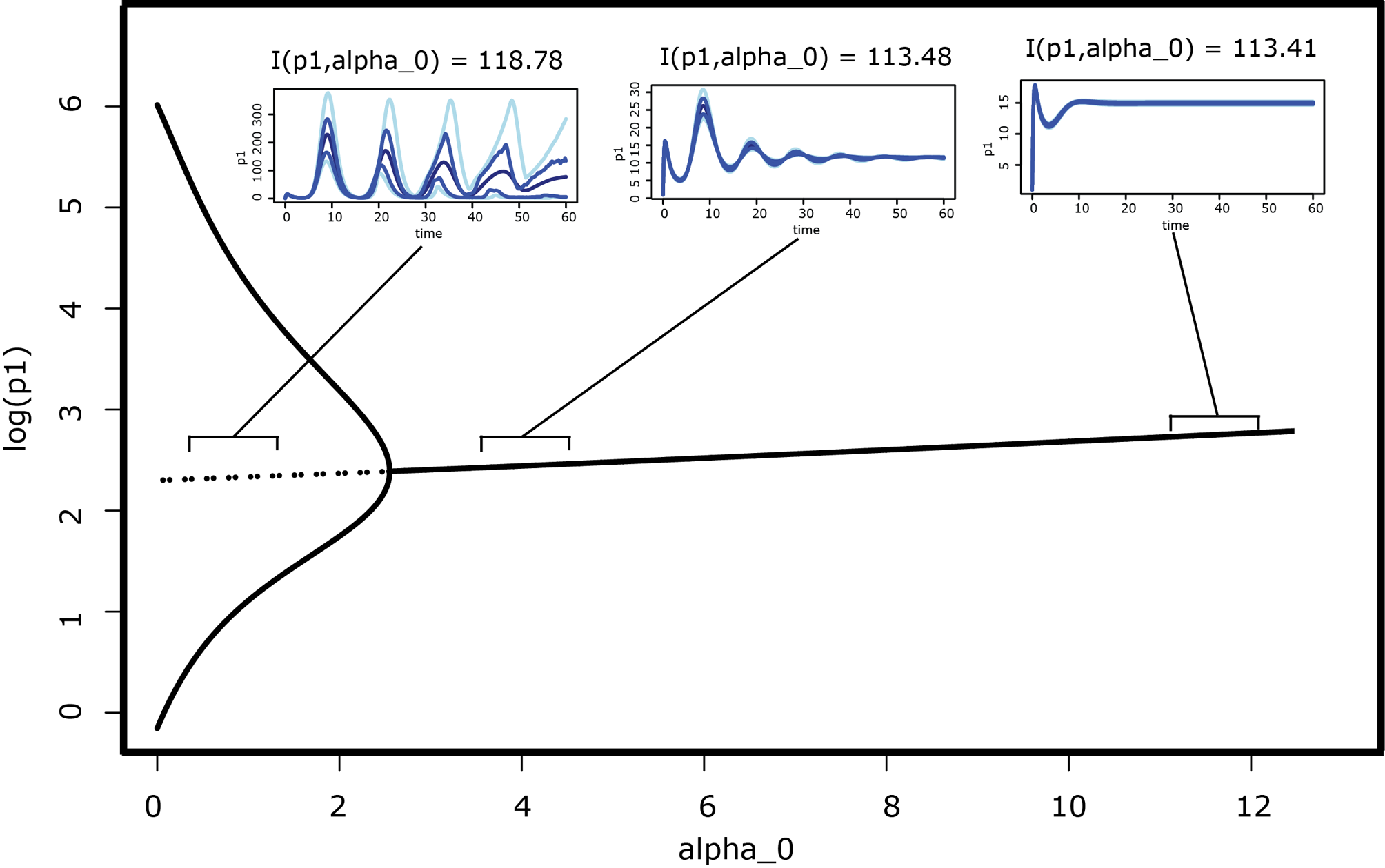

Supplement: Figure S1 — Information content of different parameter regimes. The mutual information depends on the dynamics of the system given the prior of the system parameters: the more the dynamics for different parameter values differ from each other, the higher is the information content. To visualize this we compute the mutual information between one parameter and the outcome of the system for three different regimes in the repressilator example. Noting that is a bifurcation parameter and is a Hopf bifurcation point, we choose different prior regimes for : , and . We keep the remaining parameters constant: , and . For these three priors we estimate the mutual information and represent the dynamics of the output of the system. We observe that the dynamics resulting from the first prior regime are most diverse and therefore has the highest value () compared to the remaining two parameter regimes ( for and for ). Shown is the bifurcation diagram for parameter with its stable (solid lines) and unstable (dashed lines) states. Estimation of mutual information was performed for 3 different parameter regimes. For illustration we plot the mean (dark blue), 25 and 75 percentiles (blue) and the 5 and 95 percentiles (light blue) of trajectories simulated with 10000 parameter sets, where is uniformly sampled and the remaining parameters are kept constant (, and ). (TIFF) [file pcbi.1002888.s001.tiff]

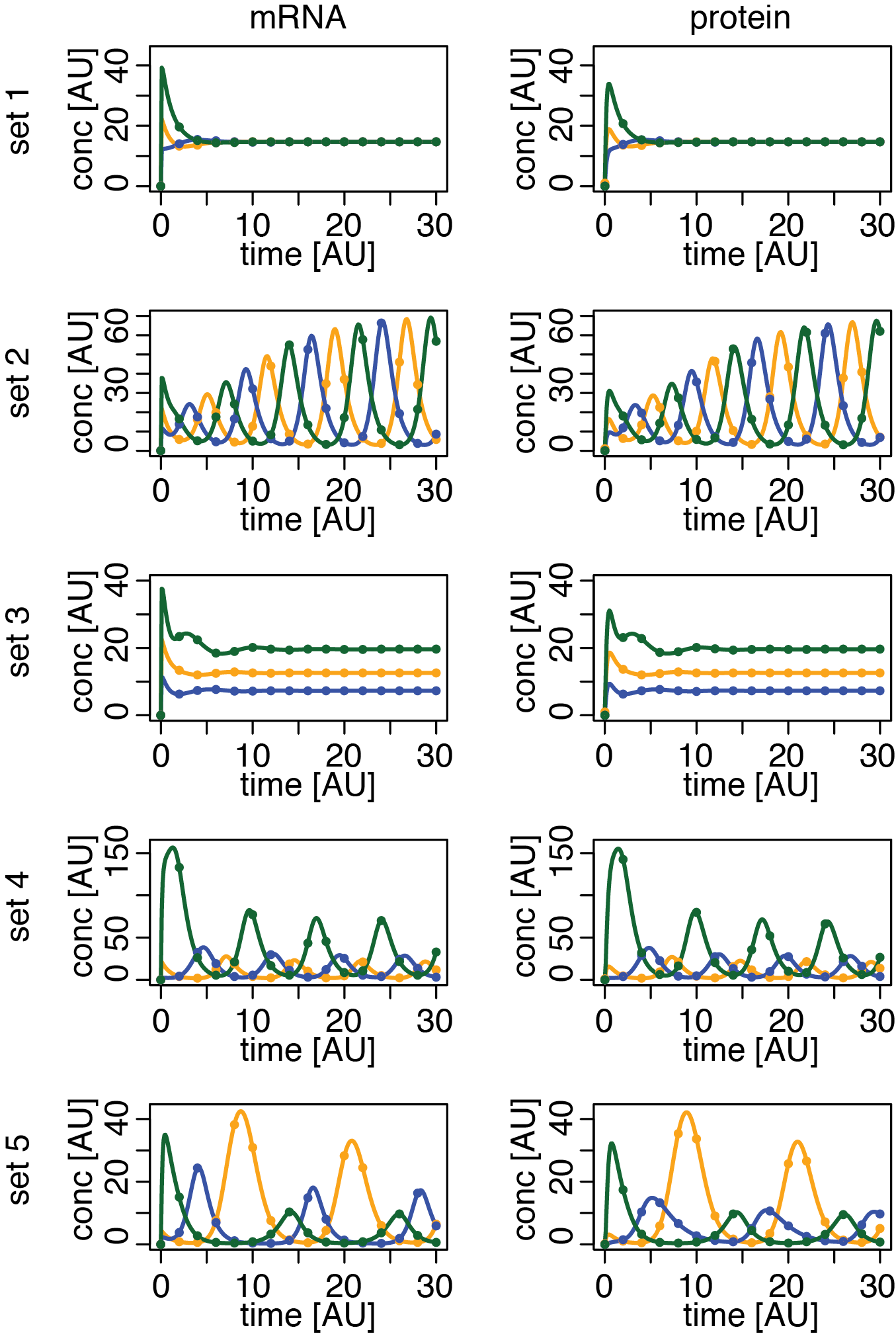

Supplement: Figure S2 — The simulated evolution of the mRNA and protein concentration in the repressilator model for each experimental setup. The parameter vector used for simulations is . The colours correspond to those in Figure 2. The dots represent the simulated data and the lines correspond to the mean of the species for parameters sampled from the posterior distribution computed using ABC SMC. (TIFF) [file pcbi.1002888.s002.tiff]

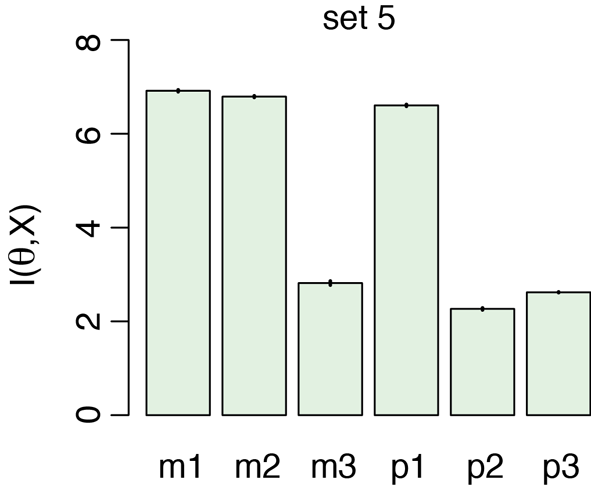

Supplement: Figure S3 — Mutual information between the parameter and each species ( mRNA and protein measurements) for experiment in the repressilator model. (TIFF) [file pcbi.1002888.s003.tiff]

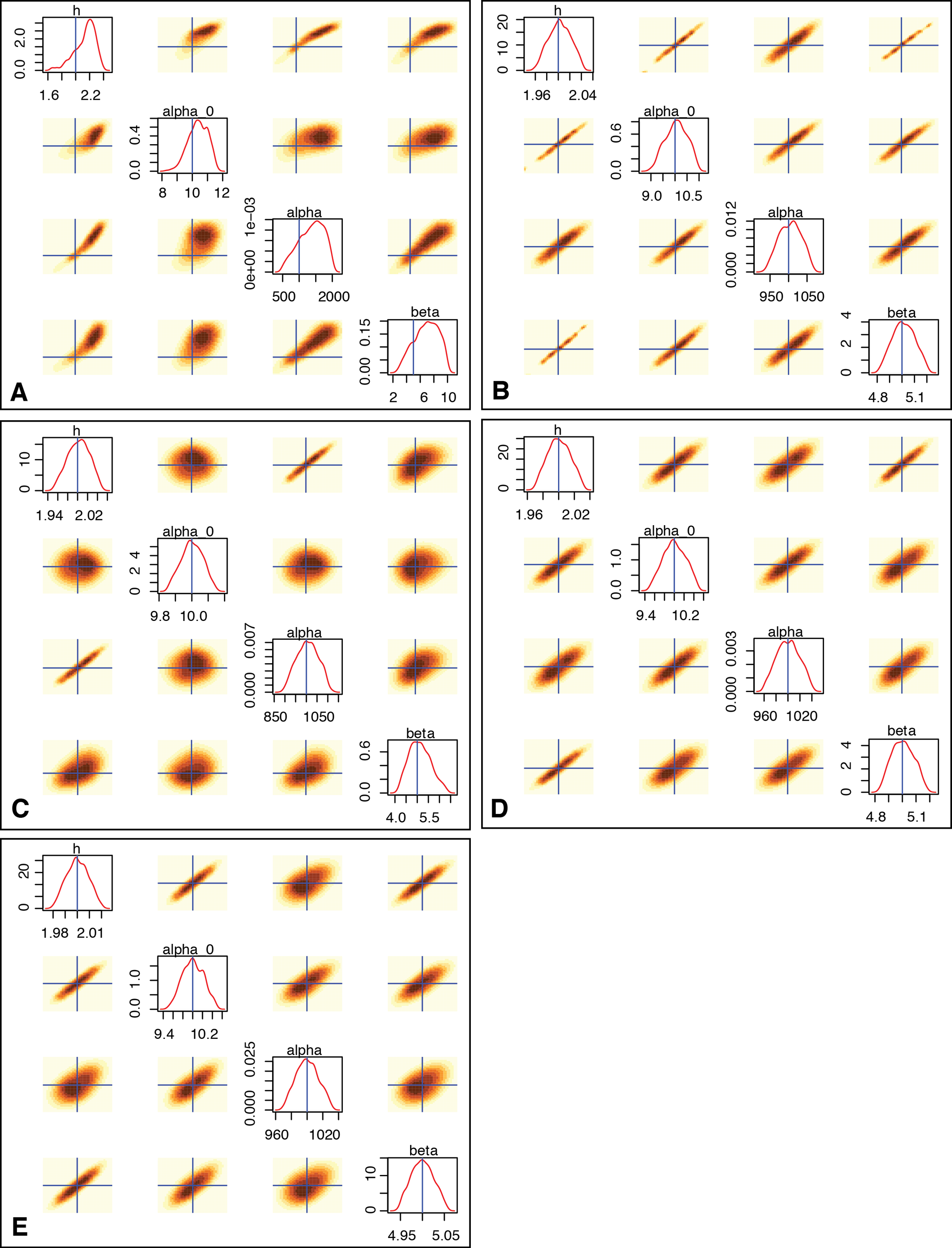

Supplement: Figure S4 — The posterior distribution given the data represented in Figure S2. Each subfigure (A to E) corresponds to an experiment ( to ). In each subfigure, the diagonal represents the marginal posterior distribution for each parameter and the off-diagonal elements show the correlations between pairs of parameters. (TIFF) [file pcbi.1002888.s004.tiff]

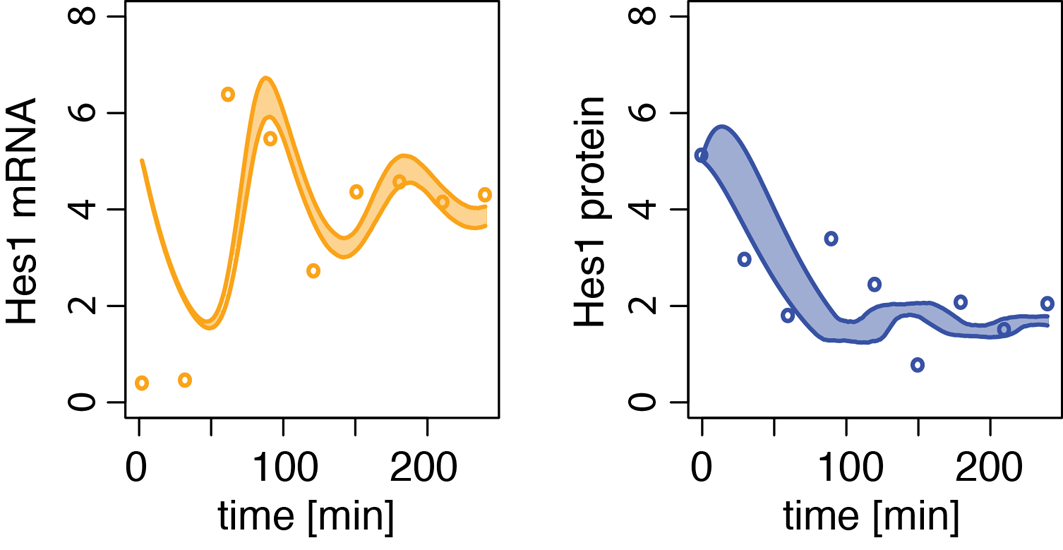

Supplement: Figure S5 — Simulated trajectories of the mRNA and protein concentrations (dots). The parameter used for simulation is The lines represent the and percentiles of the species abundances for parameters sampled from the posterior distribution computed using ABC SMC. (TIFF) [file pcbi.1002888.s005.tiff]

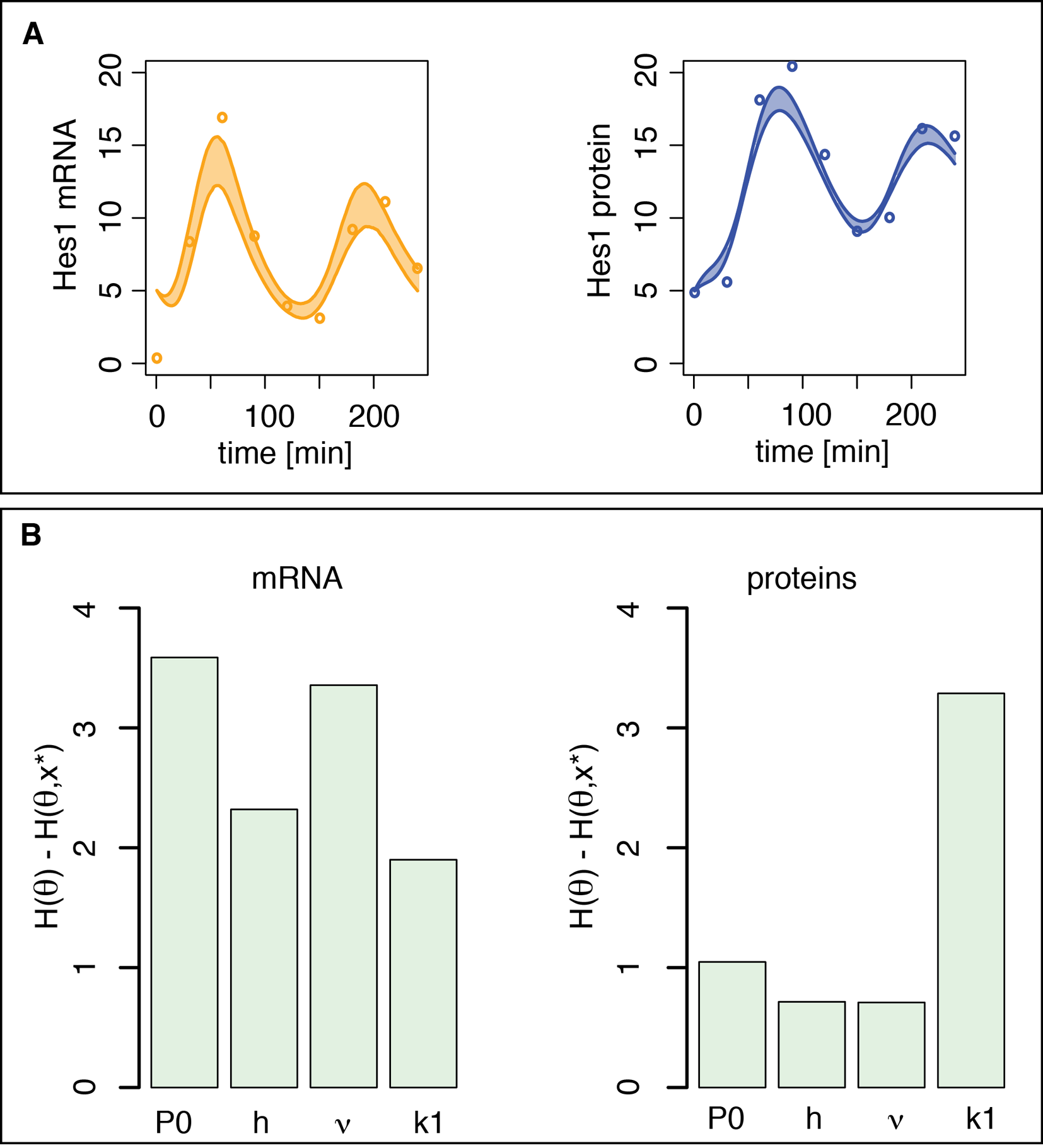

Supplement: Figure S6 — (A) Simulated trajectories of the mRNA and protein concentration (dots) for the parameter The lines represent the and percentiles of the species abundances for parameters sampled from the posterior distribution computed using ABC SMC. (B) Estimates of the differences between the entropies of the prior and posteriors. (TIFF) [file pcbi.1002888.s006.tiff]

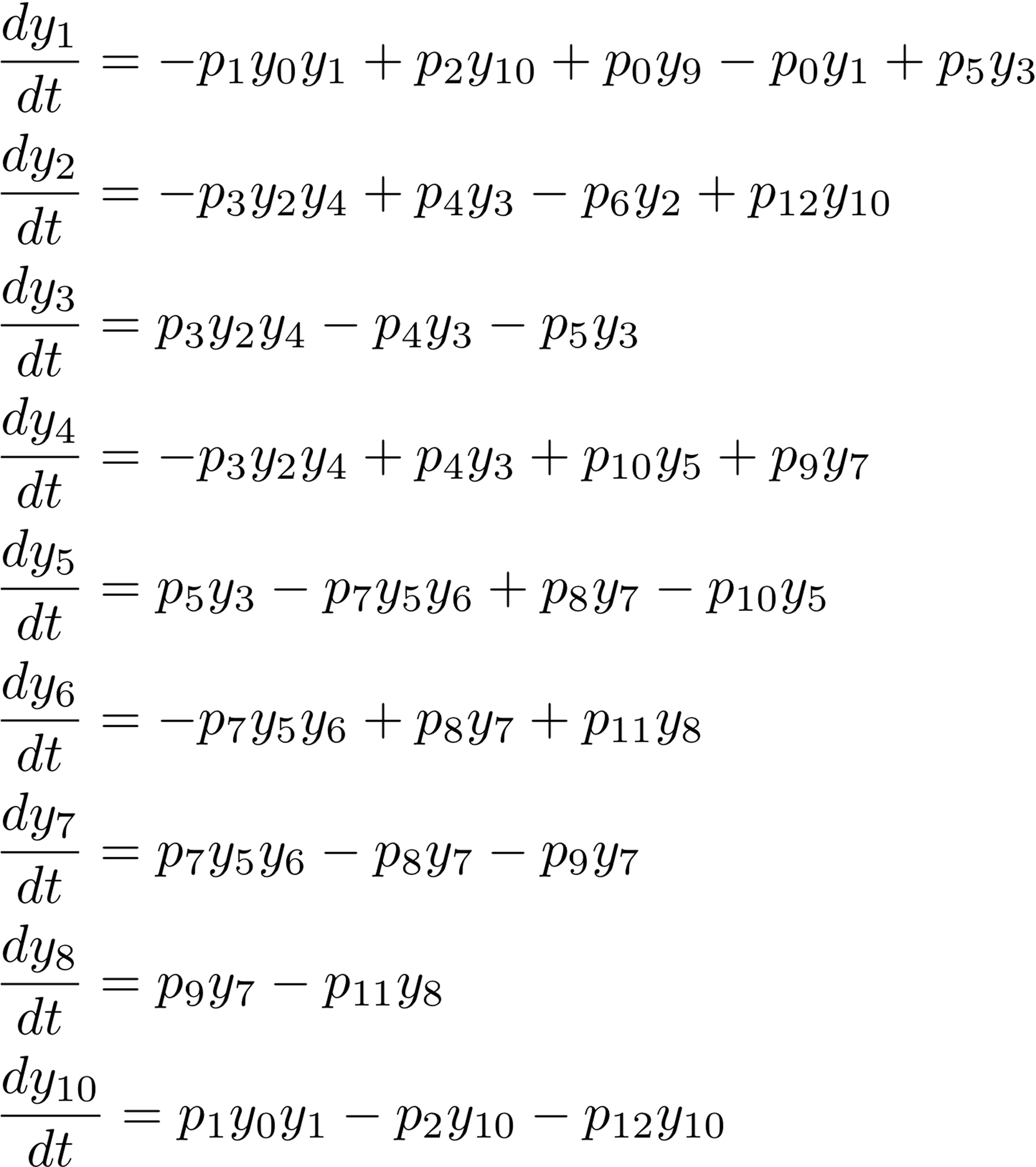

Supplement: Figure S7 — Ordinary differential equations which describe the dynamics of the species of the AKT model. The model contains parameters denoted , . The concentration of the following species (in this order) are denoted by , : EGF, EGFR, pEGFR, pEGFR-AKT, AKT, pAKT, S6, pAKT-S6, pS6, pro-EGFR and EGF-EGFR. (TIFF) [file pcbi.1002888.s007.tiff]

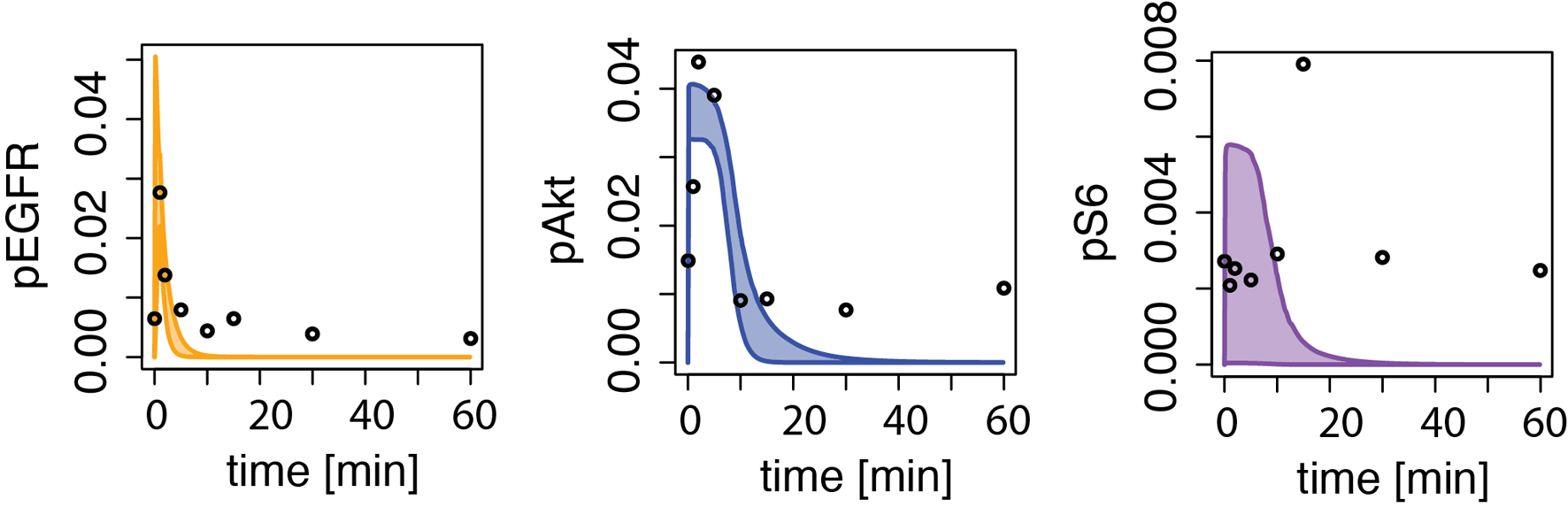

Supplement: Figure S8 — The time course of phosphorylated EGF receptor (pEGFR), phosphorylated Akt (pAKT) and phosphorylated S6 (pS6) in response to an impulse input of EGF over seconds with an intensity of ng/ml (dots). Data are Western blots measurements, described in [45]. The lines represent the and percentiles of the evolution of the species for parameters sampled from the posterior distribution computed using ABC SMC. (TIFF) [file pcbi.1002888.s008.tiff]

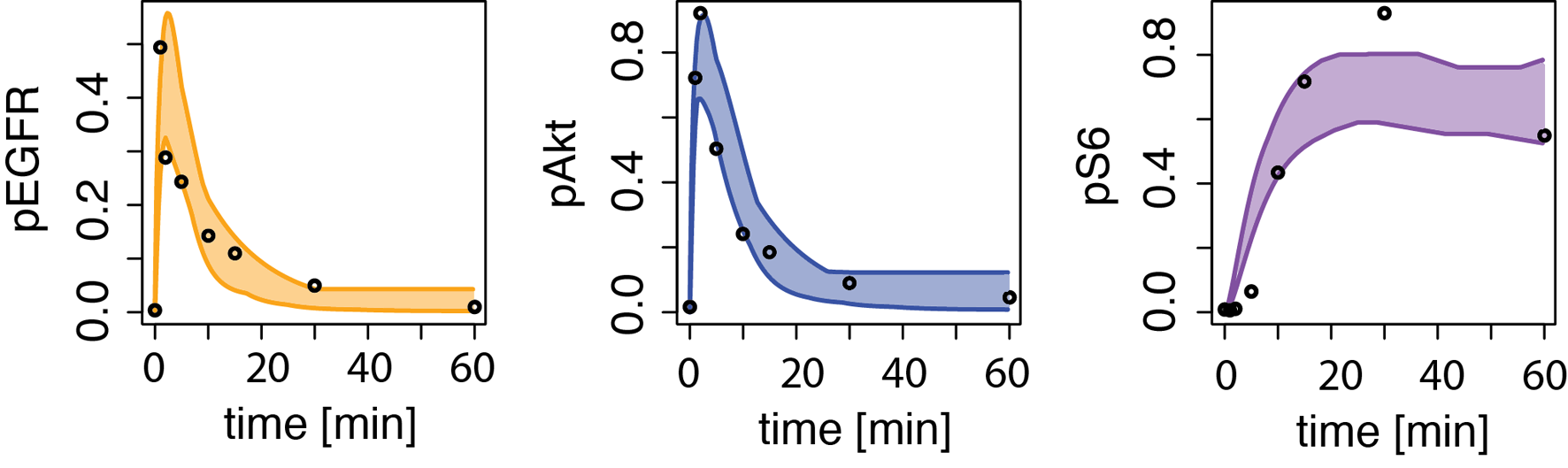

Supplement: Figure S9 — The time course of phosphorylated EGF receptor (pEGFR), phosphorylated Akt (pAKT) and phosphorylated S6 (pS6) in response to a step input of EGF over minutes with an intensity of ng/ml (dots). Data are Western blots measurements, which have been generated and published by [45]. The lines represent the and percentiles of the evolution of the species for parameters sampled from the posterior distribution computed using ABC SMC. (TIFF) [file pcbi.1002888.s009.tiff]

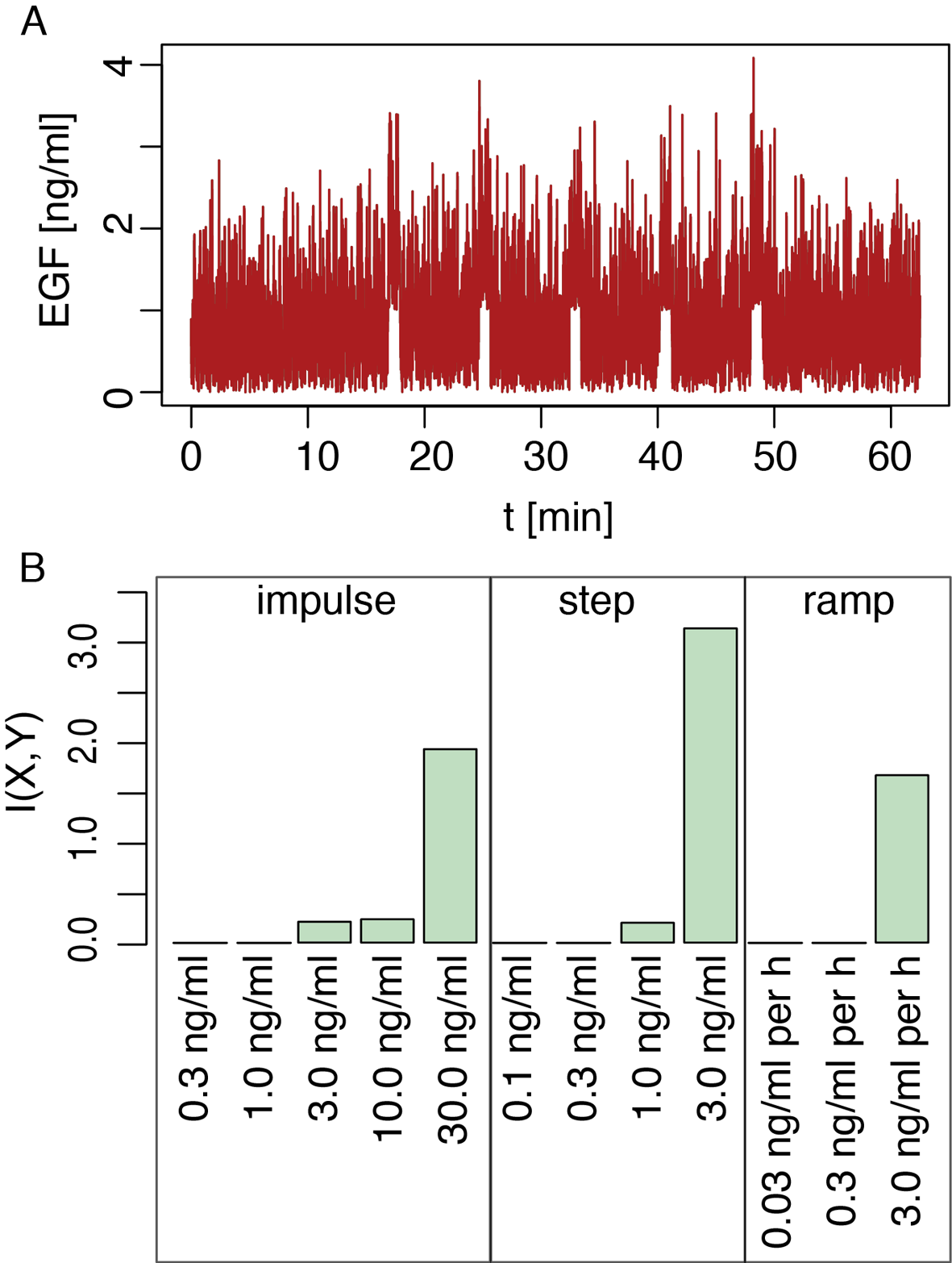

Supplement: Figure S10 — (A) A noisy -impulses EGF input signal: the pulses are of intensity ng/ml and length seconds spaced by seconds with an additive background noise which is the absolute value of a gaussian white noise of variance . (B) The mutual information between the time course of the species of interest under the noisy input signal represented in (A) and the time course of the species under each of the following possible experiments: an impulse stimulus of length seconds with possible intensity (, , , and ng/ml), a step stimulus of length minutes with possible intensity (, , and ng/ml) and a ramp stimulus of length minutes with possible final intensity (, and ng/ml). (TIFF) [file pcbi.1002888.s010.tiff]

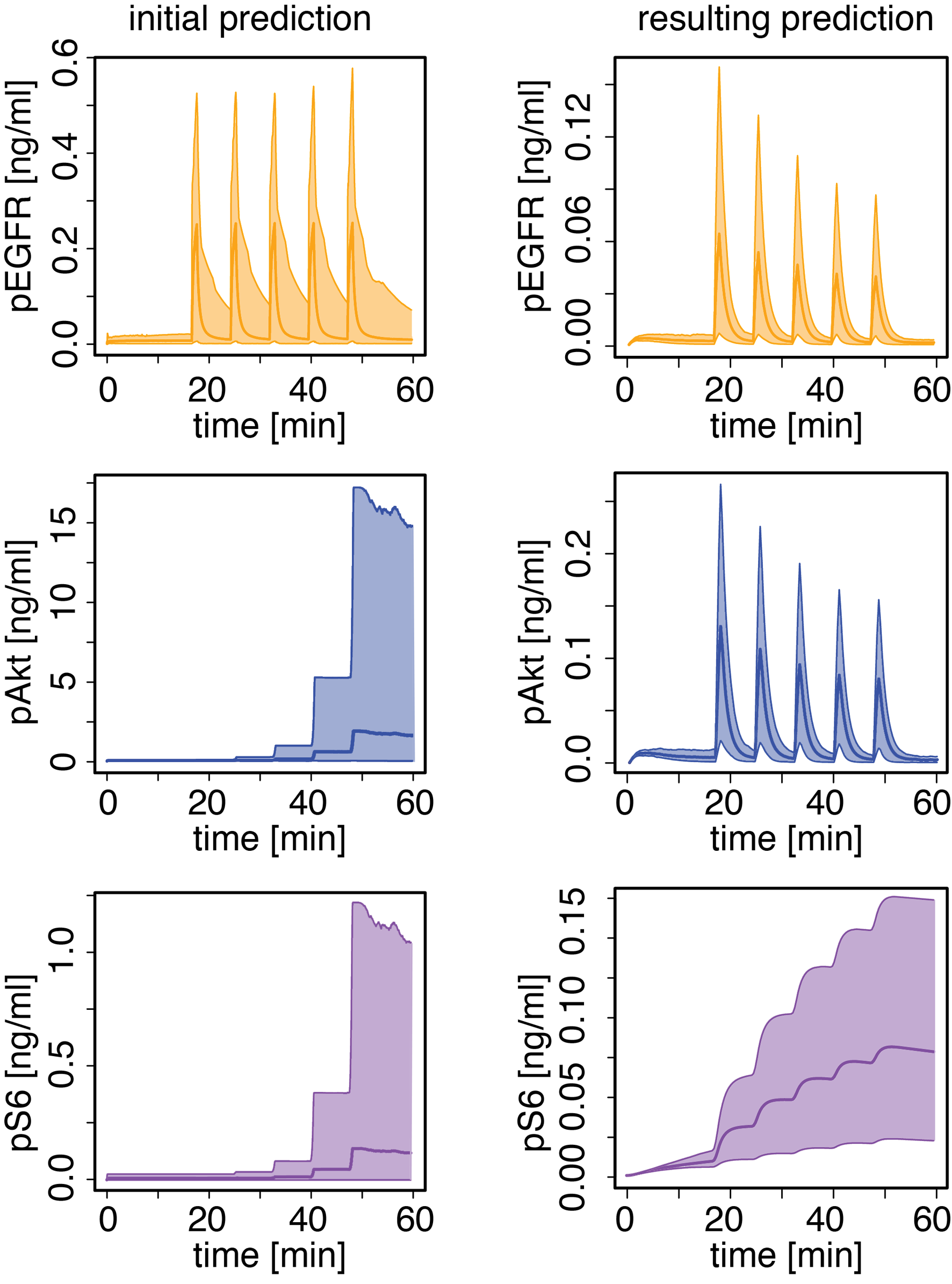

Supplement: Figure S11 — The predicted time course of the proteins pEGFR, pAKT and pS6 under the noisy -impulses EGF input signal with a noise of high intensity represented Figure S10 A. In the left panel, the prediction is based on the initial dataset whereas in the right panel in addition to the initial data it is also based on the outcome of the step stimulus with intensity ng/ml, which is the experiment with the highest mutual information. The scale of the y-axis is different for each figure. (TIFF) [file pcbi.1002888.s011.tiff]
